# Sexual abuse and risky sexual behaviors among young female hawkers in Burkina Faso: a mixed method study

**DOI:** 10.1186/s12914-016-0109-8

**Published:** 2017-01-04

**Authors:** Saide Yacine Y.A. Ouédraogo, Ebrima J. Sisawo, Song-Lih Huang

**Affiliations:** 1 National Yang-Ming University, Institute of Public Health, International Health Program, Taipei, Taiwan; 2 Direction Régionale de la Santé du Centre Est, Tenkodogo, Burkina Faso

**Keywords:** Hawker, Sexual behavior, Harassment, Bittou, Boromo, Burkina Faso, Violence, Workplace

## Abstract

**Background:**

Young street hawkers in Burkina Faso are increasingly exposed to workplace hazards such as physical and sexual abuse, and also unsafe sexual practices. The objectives of this study were to identify the socio-demographic status and work characteristics of young female hawkers, describe their sexual behavior and their experience with regards to sex-related violence at the workplace.

**Methods:**

The study used a mixed design combining qualitative and quantitative methods. It was carried out in two traffic stations in Burkina Faso namely Bittou customs station and Boromo bus station. Female hawkers aged 13 – 24 years were invited to participate in a questionnaire survey and local key informants were recruited to partake in an in-depth interview. The recruitment was based on their duties related to the hawkers.

**Results:**

The study included 264 participants in the survey and 16 interviewees. The survey showed that three quarter of participants had primary education or lower. About half of them had been sexually harassed, with clients, public members and co-hawkers as the most common source of assault. Most (68.6%) hawkers were sexually active; among them 43.7% had received money or gifts for sex. Positive factors associated with commercial sex include working in Boromo and age above 17, while negative factors include being Muslim and having female genital mutilation. The interviews confirmed the relationship between hawking and the socio-economic situation of participant’s family, and pointed out societal factors that expose hawkers to risky sexual behaviors.

**Conclusion:**

This study provides a better understanding of young female hawking activity in Boromo and Bittou. Implementing an empowerment program for female street vendors and their families, and an efficient surveillance system might help reduce these hazards.

**Electronic supplementary material:**

The online version of this article (doi:10.1186/s12914-016-0109-8) contains supplementary material, which is available to authorized users.

## Background

### Socio-economic status of women street vendors in Africa

In low income countries, informal employment is generally more common for women than formal employment. In sub-Saharan Africa, 84% of female non-agricultural workers are informally employed compared with 63% of male non-agricultural workers [1]. These women may be the main breadwinner of the family, which is often the case for women street traders [2]. The reason for entering street trading is closely related to the low socio-economic status of the women, who are often unable to get regular jobs in the remunerative formal sector because of their low level of education and skills [3]. Vulnerable groups such as illiterate or semi-literate, unskilled women and children from rural areas usually get into the informal economy, and many of them become street traders in African countries, where the majority of street traders are women: 63% in Kenya, 68% in South Africa and 88% in Ghana, a neighboring country of Burkina Faso [4, 5].

A street vendor is broadly defined as a person who offers goods for sale to the public without having a permanent selling built-up structure. Street vendors may be stationary in the sense that they occupy a particular location on the pavements or other public/private spaces, or they may be mobile and carry their goods on push carts or in baskets [6]. In Burkina Faso hawking is generally carried out by young females who are thought to be more capable than boys in attracting and approaching clients. In this study, the terms ‘street vendor’ and ‘hawker’ will be used interchangeably.

### Gender inequality and sex-related abuse among young female hawkers

In many countries, girls face double discrimination in general: by age and by gender. Unequal treatment and violence against women and girls remain a significant challenge worldwide. About two thirds of the world’s illiterate adults are women [7], as girls are more likely to miss schools since they are often required to help in house chores or earning money. Many traditional cultures consider child labor as part of the child’s socialization process and it is acceptable for children, especially girls, to start vending in the street at a very young age [8].

Young girls working as street vendors in open and crowded places are vulnerable to violence and sexual abuse. It has been established that young women’s susceptibility to sex-related injury is much higher than that of their male counterparts [9]. This may include verbal and physical abuse, sexual manipulation and domination, forced sex or forced sexual practice [10]. Sexual abuse is associated with a range of subsequent health problems, including injuries, HIV and other sexually transmitted diseases, unwanted pregnancies, abortion and its complications and mental health problems.

### High HIV prevalence among young girls

Worldwide, HIV/AIDS prevalence is highest among young girls. In 2013, almost 60% of all new HIV infections occurred among adolescent girls and young women aged 15–24 [11]. Biological factors, lack of access to information and health services, economic vulnerability and unequal power in sexual relations create a foundation for the spread of HIV/AIDS among them. Young female hawkers are particularly afflicted by these factors since hawking is associated with poverty, economic and social inequality [12]. Clear links have been established between female street hawking and the transmission of HIV/AIDS [13–15]. Since 2001, female street hawkers have been identified as a priority and specific vulnerable group in Burkina Faso [16]. Indeed the prevalence of HIV/AIDS was estimated at 13.1% among female street vendors by “Programme d’Appui au Monde Associatif et Communautaire” (PAMAC) during a screening campaign in 2006, similar to that of sex workers (12.5%) [17].

Studies focusing on reproductive health and sexual risks among young female hawkers in Sub-Saharan Africa are rare. This is partly because of the limited accessibility of the target population due to its high level of mobility [18, 19]. Furthermore, current literature concerns mostly street vendors in urban area; very few have explored hawking in rural areas. To the best of our knowledge, most studies undertaken in West Africa used cross-sectional quantitative method. To obtain a more comprehensive picture of hawkers’ situation, and to know if local government or non-governmental organizations have taken any concrete measures to ensure their safety and health, the current study interviewed local key informants whose duties involve dealing with hawkers on a daily basis. One final point to make: female genital mutilation (FGM) is common in Burkina Faso with a national rate of 75.8% in 2010 among women aged 15–49. The Central East region had the highest rate of 90% and Bittou is in this region [20]. Since one of the purposes of this procedure is to inhibit female sexual behavior, it is meaningful to know the prevalence of FGM among female hawkers and see if it is associated with any of the sex practices. This study attempts to address these research gaps and aim to (1) identify the socio-demographic status and work characteristics of young female hawkers, describe their sexual behavior and occupational hazards including sex-related violence; (2) describe the perception of and reaction to hawking from the viewpoints of informants in local agencies.

## Methods

### Study area

This research was conducted in two traffic stations; one situated in Boromo and the other in Bittou, all in Burkina Faso. The two traffic stations are characterized by sheer number of street vendors. The Boromo bus station is located on the main national road that connects the two largest cities of the country. Bittou is a small town close to a point that borders Burkina Faso with two countries, namely, Ghana and Togo. Bittou has one of the largest customs offices in the country, where trucks and vehicles stop over for customs transactions and paper works. These two stations are populated by many street vendors, mainly female of all ages.

### Study design

This study used a mixed design, combining a qualitative method (key informant interviews and field observations) and a quantitative questionnaire survey [21]. Mixed method was used to acquire a fuller understanding of hawking activity by combining the information collected from hawkers themselves and key informants who have close relationships with hawkers. The interviews with local key informants provide insight into the household characteristics of hawkers, the community’s views on hawking and hawkers, and the actions taken by their agencies to help hawkers.

### Sampling procedure and sample size

Given that there was no existing list of street hawkers, we used convenience sampling to recruit participants for the questionnaire survey, similar to other studies on the same population [10, 22, 23]. The study included female street vendors aged 13–24 years hawking in or around the selected bus stations. Vendors in markets or those who declined the interview were excluded. Of those approached, most (with very few exceptions) agreed to participate and the final sample size was 264 (134 in Boromo and 130 in Bittou). On the fifth day of each field study we found that most of the eligible hawkers present at the site during the data collection period had been interviewed.

### Data collection

The data were collected at each site by two well-trained female research assistants; one from Bittou and the other from Boromo. The research assistants have fluency in French as well as the two most common local languages. The survey was performed using a face-to-face interview with a pretested semi-structured questionnaire (Additional file 1). This questionnaire was written in French and the interviewer translated questions in the local language where necessary.

The qualitative investigation involved 16 local key informants in total (7 in Bittou and 9 in Boromo). Interviewees were local officials, members of local associations and traditional chiefs and former hawkers. Participants were selected purposively in each locality according to the connection they had with hawkers. Respondents were alone with the researcher during the interview. All interviews were audio-recorded in addition to the notes taken by the researcher. Each interview lasted for about 20 –30 min. Questions asked in the interview included their personal opinions on hawking, the reasons why young females were involved in hawking, problems associated with hawking and measures their agencies had taken to deal with negative consequences.

### Data analysis

We performed descriptive analyses, Chi-square tests and multivariate analysis to see the associations between variables using Stata 13. For all the tests we considered a significance level of 0.05. Regarding the qualitative part, responses from the interview were transcribed in French and then translated into English. Textual analysis was performed by first identifying the key words and sentences, and then recurring ideas were grouped to construct codes, then themes. The categorization was repeatedly examined by the investigators for coherence and consistency.

### Ethical consideration

An approval of the study was obtained from National Ethics Committee of Burkina Faso before data collection. In addition, formal letters seeking permission were submitted to the Ministry of Social Welfare, the Ministry of Women’s Affairs and to mayors of cities where the study was conducted. Before starting conversations for both the survey and the in-depth interview, verbal informed consent was obtained from the participants after the purpose of the study had been clearly explained to them. Written informed consent was waived because most of the participants were not able to read French. Principles such as confidentiality of the information collected and total anonymity of the participants were respected in the conduct of the study. Each interview was conducted in private and there was no name or identifiers on the questionnaire. The data is stored safely by the principal investigator of the study.

## Results

### Quantitative result

#### Socio-demographic characteristics

Table 1 summarizes the socio-demographic characteristics of participants by site and the comparison between Boromo and Bittou. Participants aged between 13 and 24 years (mean 18 ± 3.37) and most of them were single. Many female hawkers, 38.5% in Bittou and 53% in Boromo, had never attended school and few had reached high school. In addition, 35.2% of the total respondents were still enrolled in schools.

**Table 1:** Socio-demographic characteristics of participants by site

Variables	Bittou		Boromo		Total		*p*-value
	*n*	%	*n*	%	*n*	%	
Range of Age							
13–16	52	40	48	35.8	100	37.9	0.757
17–20	46	35.4	49	36.6	95	36	
21–24	32	24.6	37	27.6	69	26.1	
Marital Status							
Single	115	88.5	96	71.6	211	79.9	<0.001*
Married	7	5.4	19	14.2	26	9.8	
Concubine	5	3.8	17	12.7	22	8.3	
Separated/divorced	3	2.3	1	0.7	4	1.5	
Other	0	0	1	0.7	1	0.4	
Educational Level							
Not attend school	50	38.5	71	53	121	45.8	0.033*
Primary	23	17.7	31	23.1	54	20.5	
Secondary school	40	30.8	31	23.1	71	26.9	
High school	8	6.2	1	0.7	9	3.4	
Informal education	9	6.9	0	0	9	3.4	
Current status							
Studying	53	40.8	40	29.8	93	35.2	0.063
Not Studying	77	59.2	94	70.2	171	64.8	
Religion							
Muslim	104	80	102	76.1	206	78	0.203
Catholic	12	9.2	21	15.7	33	12.5	
Protestant	13	10	8	6	21	8	
Other	1	0.8	3	2.2	4	1.5	
Been sexually mutilated							
No	60	46.1	44	32.8	104	39.4	0.027*
Yes	70	53.9	90	67.2	160	60.6	

* = Statistically significant at *P* < 0.05

With regards to religion, Muslims were predominant in both sites. The large part of young female hawkers, particularly those in Bittou, was still living with their parents who were mostly illiterate and were likely to be farmers or petty traders. More than half of participants had undergone female genital mutilation (FGM), and the prevalence was higher in Boromo.

#### Work characteristics and conditions

With regard to hawking activity, most (63.8% in Bittou and 80.6% in Boromo) hawkers were selling almost every day, and the majority of them reported being self-employed (63.3%) or hawking for the family (31.4%). On average the mean age of getting into hawking activity was 12 (±3.45) years old and nearly a third started regular hawking before the age of 10. They work very long hours, with 68.9% reported 8–12 h, and 24.3% reported longer than 12 h, particularly in Boromo. In addition, 5.4% in Bittou and 15.7% in Boromo said they sometimes stayed at the workplace overnight.

#### Sexual abuse

Sexual harassment was commonly encountered among hawkers, as half of the total participants reported having been sexually harassed at workplace (Table 2). Physical and verbal abuse was very prevalent in both sites. Six (9.1%) cases of rape were reported in Boromo and none in Bittou. Perpetrators were commonly clients in Bittou (83.6%) and male co-hawkers in Boromo (56.1%) followed by unknown public members in both sites.

**Table 2:** Sexual harassment experienced by hawkers by site

Variables	Bittou		Boromo		Total		*p*-value
	*n*	%	*n*	%	*n*	%	
Ever being sexually harassed							
No	63	48.5	68	50.7	131	49.6	0.710
Yes	67	51.5	66	49.3	133	50.4	
Type of Harassment^†^ (*n* = 133)							
Verbal	49	73.1	50	75.8	99	74.4	0.729
Visual	15	22.4	25	37.9	40	30.1	0.051
Physical	54	80.6	31	47	85	63.9	<0.001*
Rape	0	0	6	9.1	6	4.5	0.012*
Other	27	40.3	10	15.1	37	27.8	0.001*
Assaulter^†^ (*n* = 133)							
Client/Customer	56	83.6	29	43.9	85	63.9	<0.001*
Public member	38	56.7	30	45.5	68	51.1	0.194
Co-hawker	24	35.8	37	56.1	61	45.9	0.019*
Driver	36	53.7	17	25.8	53	39.9	0.001*
Apprentice	35	52.2	16	24.2	51	38.4	0.001*

^†^ = Multiple Coding variables * = Statistically significant at *P* < 0.05

#### Sexual behavior of female hawkers

Table 3 shows the sex practices of participants. Overall, more than two thirds of young female hawkers were sexually active. Among them, a third had sexual intercourse before the age of 14. Overall, 77 (42.5%) had sex with more than two sexual partners within 3 months of the study. However, more than 70% reported not using condom during their last sexual intercourse. Those who had two or more sexual partners were not more likely to use condoms. In parallel with the lack of protection, almost half (45.3%) of participants reported at least one unplanned pregnancy in their life. One particular risky behavior for hawkers was engagement in commercial sex. A significant number of them reported receiving money or gifts in exchange for sexual intercourse, and the prevalence was higher in Boromo. We examined to see if FGM was associated with any of the sex practices, since the purpose of this painful procedure is to inhibit female sexual behavior. There was no significant association between FGM and being sexually active. In addition, FGM was not statistically associated with the following factors, including religion, age of sexual initiation, number of sexual partners, sex in exchange for money or gifts, and condom use.

**Table 3:** Sexual behavior of participants and their knowledge on HIV/AIDS by site

	Bittou		Boromo		Total		*p*-value
	*n*	%	*n*	%	*n*	%	
Sexually active							
No	48	36.9	35	26.1	83	31.4	0.059
Yes	82	63.1	99	73.9	181	68.6	
Age at the first sexual intercourse (*n* = 181)							
14 years or younger	21	25.6	39	39.4	60	33.2	0.051
15 years or older	43	52.4	49	49.5	92	50.8	
I don’t remember	18	22	11	11.1	29	16	
Number of sexual partner during the past 3 months (*n* = 181)							
1 person	48	58.5	53	53.5	101	55.8	0.168
2 to 4 people	26	31.7	41	41.4	67	37	
5 people or more	5	6.1	5	5.1	10	5.5	
No sex in past 3 months	3	3.7	0	0	3	1.7	
Condom use during the last sexual intercourse (*n* = 181)							
No	47	57.3	81	81.8	128	70.7	<0.001*
Yes	35	42.7	18	18.2	53	29.3	
Ever get unwanted Pregnancy (*n* = 181)							
No	50	61	49	49.5	99	54.7	0.122
Yes	32	39	50	50.5	82	45.3	
Ever received money or gift for sexual intercourse (*n* = 181)							
No	43	52.5	41	41.4	84	46.4	<0.001*
Yes	22	26.8	57	57.6	79	43.7	
Proposed but I refused	17	20.7	1	1	18	9.9	
Ever heard of HIV/AIDS							
No	13	10.0	18	13.4	31	11.7	0.388
Yes	117	90.0	116	86.6	233	88.3	

* = Statistically significant at *P* < 0.05

Logistic regression was used to examine the factors associated with receiving incentives for sex (Table 4). We found the following factors to be associated with higher likelihood: older age, working in Boromo and being catholic (compared to Muslim). Finally, girls who had undergone FGM were less likely to practice commercial sex (AOR = 0.46; 95% CI: 0.24–0.94).

**Table 4:** Multivariate logistic regression analysis results showing association between ever received money or gift for sex and selected variables among socio-demographic characteristics of participants (*n* = 264)

Variables	COR (95% CI)	AOR (95% CI)
Site		
Bittou	1	1
Boromo	3.33^*^ (1.87–5.95)	4.42^*^ (2.21–8.85)
Age Range		
13–16	1	1
17–20	3.22^*^ (1.6–6.49)	3.85^*^ (1.73–8.54)
21–24	5.62^*^ (2.67–11.82)	12.22^*^ (4.68–31.93)
Marital status		
Single	1	1
Married/Concubine	1.4 (0.72–2.72)	0.42 (0.18–1.02)
Religion		
Muslim	1	1
Catholic	4.56^*^ (2.09–10.02)	5.6^*^ (2.28–13.78)
Protestant	1.92 (0.69–5.32)	2.84 (0.87–9.21)
Other	5.5 (0.49–61.85)	6.05 (0.46–80.27)
Having FGM		
No	1	1
Yes	0.85 (0.5–0.82)	0.46^*^ (0.24–0.94)

^*^Statistically significant at *P* < 0.05

### Qualitative results

In addition to the survey, we carried out a qualitative investigation through an in-depth interview with local authorities and key informants. This approach was used in order to provide a better understanding of the hawking phenomenon and its consequences. These key informants had close contacts with street vendors and described hawkers’ situation from various points of view.

#### Field observation: workplace environment

Boromo station was a large and busy open place surrounded by stores and restaurants. The station was noisy and crowded both day and night. Hawkers across all ages could be seen, particularly young girls. Inside the station, they were seen carrying trays on their heads or transportable tables on which they display their merchandize (vegetables, fruits, food, drinks, cookies etc.) and go from bus to bus, trying to reach passengers and drivers. Boromo’s station was both a business place and an entertainment area, with a bar on the second floor of the centrally located station building. The place was lively all the time (24 h seven days a week) but no formal regulation was noticeable. The station was the responsibility of Boromo mayor’s office, which was located one or two kilometers away. According to the mayor, many attempts to organize the station had been tried by the municipality but were unsuccessful. He said: “*Currently there is neither security nor management committee at the station. Anytime we tried to set up a committee or security guards there, the first week it works well, but after several days, it fails”*.

Bittou customs station was a much larger open space where there were numerous parked vehicles (trucks and cars) surrounding the customs office. Here, like Boromo bus station, hawkers’ populations were predominantly young girls and some women. There were also no regulation or security settings. Female hawkers usually come from home with their merchandizes in a tray or basket which they carry and dodge between parked trucks and cars to sell to drivers and their apprentices. They also vend to brokers and some passengers. All these female hawkers interacted constantly with their clients.

#### Societal and family factors related to female hawking

In both Bittou and Boromo, key informants pointed out that by vending on the street, women and their daughters contribute to their families’ income, but this could make young girls violence-prone. The Women’s Promotion Coordinator in Boromo explained: “*Young girls stay with their mothers at the station until late in the evening and when they return home, the head of the family who is usually left alone in the house don’t say anything because he deems the petty cash generated from hawking as rewarding.”*

Hawking activity is passed from one generation to the next. Indeed, many girls grew up in the hawking environment, particularly in Boromo because it is the main activity of most women who eventually pass the trade onto their daughters. A member of an association in Boromo reported: “*When you go to the bus station, everyday you see babies there. Their mothers put them in cartons under tables and go about selling. These babies are being raised there and, when they grow up they start helping their mothers on hawking activities and then end up staying at the station as vendors and also have children there. So it’s a vicious cycle”*.

Most respondents agreed that hawking by young females was primarily associated with the poor socio-economic position of their families. Families in the study were mostly Muslims and polygamous; usually each mother is responsible for providing her own children. A former hawker in Bittou gave the reason why many young girls were found in this activity. She said: “*Families here are polygamous and fathers cannot take care of all their children…”* she continued, *“Mothers are the ones responsible for almost everything in the household. She is obliged to give money to her daughters to buy small stuffs and resell. The benefits gained might be used either for family need or for the daughter’s needs*”. As explained by an association member: “*schools in Boromo are filled mainly by children of civil servants from other regions who are appointed by government to come and serve here, because local people prefer to send their children to the station to sell rather than put them in schools*”. Some children tried to combine schooling and hawking in the beginning but gradually abandoned schools.

#### The vulnerability of girls in the hawking activity

Key informants emphasized that vending in stations lead to many risk for hawkers. According to them, very young girls were affected by sexual abuse, as reported by Ali, a member of an association (AFSDB) in Bittou: “*Rape cases are very common here in Bittou especially among children who are the most vulnerable, ignorant and naïve”.* In case of sexual abuse such as rape, many families worry more about an eventual pregnancy rather than other consequences, and abuses without significant physical harms or pregnancies might go unattended. Indeed, a story related to contested pregnancy resulting from a rape case was collected in Bittou. The head of social welfare office in Bittou related: “*This concerns a 12–13 year old girl whose father was dead and the mother was infected with HIV and therefore couldn’t meet the needs of her daughter. So the girl was selling oranges at the customs post in order to cope. In this practice, she visited a tailor next to the station … who sexually abused her. This was followed by a pregnancy, but the tailor denied it. The girl’s family brought the issue here and we confronted the tailor who eventually admits the act, but he and the girl’s family decided to solve it amicably at home. Several months later we saw the girl with her oranges, but she was no more pregnant.”*

In the opinion of some participants, many female hawkers were engaged in commercial sex, combining it with the vending activity to raise more income. They asserted that many hawkers use vending as a cover-up for prostitution. A member of the local association, called “Jonction des Actions pour Faire Avancer l’Afrique” (JAFAA), in Boromo was very explicit on this: “*With the small merchandise they sell during the day, they still manage to maintain healthy accounts. So there is something more that maintains them there and it's probably prostitution because there are many temptations at the station*.” Another interviewee indicated that some vendors accompany political figures and businessmen on their tours to cities as a way of gaining access to the upper class. This hidden part of the hawking activity exposes hawkers to sex-related health problems.

In summary, local officials in both locations only intervene when they are called upon to help; attempts to set regulations on hawking were generally weak and short-lived. Local associations tried to provide training and educational programs but the hawkers benefit less from these activities. They instead find the daily income generated from hawking more rewarding as it provides livelihood to them and their families. In Bittou, the female vendors were viewed as poor workers burdened by household needs, while in Boromo there was additional concern on the part of interviewees about the sexual exploitation of hawkers.

## Discussion

### Socio-demographic characteristics

Both the interview and the survey pointed out the disadvantaged socio-economic conditions of vendors’ families, including low income, low education level and large family size. Street vendors in our study were relatively young, which was similar to studies in Ethiopia [24] and Nigeria [22, 25]. Being young in the streets, they can easily be taken advantage of and become victims of sexual harassment or lured into unsafe sexual practices.

For both sites, almost half of the respondents were uneducated; only 54.2% of them had at least a primary level of schooling or had attended informal education. It is very low compared to the national school attendance rate in Burkina Faso which was 83.1% between 2012 and 2013 [26]. This can be explained by the fact that this study was conducted in a rural area and only among girls; two risk factors for low enrollment and high dropout [27].

We found that 60.6% of hawkers had undergone FGM. This prevalence is lower than the national rate in Burkina Faso which was 70.3 and 89.5% among women aged between 14 and 49 years in 2010 in these two regions respectively [20]. This difference can be explained by the lower age of our study participants, or under-reporting. However, we found that FGM was not limited to the Islamic religion as suggested by some studies [28, 29]. Despite the widespread belief that FGM preserves a girl’s virginity, protects her from becoming promiscuous and prevents her from engaging in immoral behavior [30], we did not find significant association between FGM and being sexually active or having more than one sexual partner.

### Work characteristics and conditions

Long working hours, precarious working conditions and high risk of harassment, together may qualify street vending as “hazardous work” which is described by the International Labor Organization (ILO) as labor that jeopardizes the physical, mental or moral well-being of a child, either because of its nature or because of the conditions in which it is carried out. The characteristics of most of our respondents also fit the definition of child labor by ILO [31]. More attention should be given to street vending, so that their trade can be organized and regulated. It is also important to empower the parents so that they spare young girls from hawking.

### Sexual abuse

Verbal harassment was the most frequent, followed by physical harassment which includes unwanted touching, unwelcome kissing and attempt to rape. This agrees with the findings in the Baseline Survey Delhi 2010 which shows that verbal harassment was the most common form of sexual harassment [32]. Only six cases of rape (2.2% of all respondents and 4.5% of those who had suffered from sexual abuse) were reported in Bittou. This proportion is lower compared to the findings in other studies in Ethiopia and Nigeria, with a rate of approximately 15% [24, 33]. In our case, the prevalence of rape might be underestimated since key informants revealed that rape was not uncommon, and victims might be reticent to report.

### Sexual behavior

Similar to existing literature, this study revealed that female street vendors’ engagement in risky sexual behavior was very high [34–36]. These risky sexual practices include early sexual debut, multiple sexual partners, low rate of condom use, and engagement in commercial sex. In the case of hawkers in Bittou and Boromo, many interviewees implied that a lot of female street vendors were combining vending and prostitution, and the suspicion was mirrored by the survey. The samples in the current study were from two towns, and we demonstrated some difference between the two sites. Specifically, we found that female street vendors in Boromo were more likely to undertake risky sexual behaviors than those in Bittou. This can be explained by the fact that, as observed in the fieldwork and interviews, there were much more business and entertainment activities in Boromo, and hawkers there were exposed to more temptations and had less control over their working environment because they had to work late or stay at the station overnight.

We found the following factors to be associated with higher likelihood of practicing commercial sex: older age, working in Boromo and being catholic (compared to Muslim). As one would expect, elder hawkers are more exposed to coital activity. They also have more financial needs and therefore are at higher risk of engaging in commercial sex. Muslim families, especially the fathers, may be more rigorous and strict than Catholics when it comes to sexual behavior of their children.

Finally, hawkers who experienced FGM were less likely to engage in commercial sex. This association may be explained firstly by the long-term complications associated with FGM including severe dyspareunia, pelvic inflammatory disease, trauma and hemorrhage [37] and make sexual intercourse uncomfortable or painful; secondly by the cultural practices behind FGM, such as religious belief, rather than FGM itself, that made the difference. So even when it seems that FGM is associated with less commercial sex, more investigation is necessary to dissect the other factors contributing to this association.

In general the interviews and survey data complement each other. Indeed, both qualitative and quantitative data showed the relationship between hawking and socio-economic situation of hawkers’ families. With regard to sexual harassment, the risk of rape was stressed more by the interviewees but under-reported in the survey. On the other hand, the survey provides evidence for other less observable forms of harassment including visual and verbal ones. The field observation of the working settings allows us to understand why Boromo hawkers were more likely to adopt risky sexual behavior than Bittou hawkers. The two data also pointed out the link between female hawking activity and risk of taking on unsafe sexual behavior such as prostitution.

According to the Convention on the Rights of the Child, together with the Beijing Declaration and Platform for Action, every girl has the right to education. They have the right to be protected from work that threatens their health, education or development and the State shall regulate working conditions. In addition, girls have the right to enjoy the highest attainable standard of physical, social and reproductive health [38, 39]. States parties shall take all appropriate measures to implement and promote these rights.

### Limitations

The study has some limitations: first of all, it is cross-sectional so it is difficult to make a causal relationship. In addition, the results might not be generalizable to all hawkers in the country since hawking settings and places differ as seen between the two sites in this study. Another constraint of the generalization is the non-random sampling strategy used for participant recruitment. Furthermore, many questions, especially those related to sexuality, were very sensitive and were often seen as unmentionable or taboo in Burkina Faso society, therefore reporting and social desirability biases might come into play. Part of these shortcomings could be compensated by field observation and key informant interviews. However, key informants’ statements were personal judgments towards hawkers and might be subjective and not based on substantial evidence.

## Conclusion

This study allowed us to have a better understanding of young female hawking activity in Boromo and Bittou. Both qualitative and quantitative data showed the link between hawking and low socio-economic situation of the family, requiring young females, including children, to work in hazardous conditions. With regard to sexual harassment, the risk of rape was stressed more by the interviewees but under-reported in the survey, although the survey provides evidence for other forms of harassment which were visual and verbal. Our data also pointed out the link between hawking activity and risk of taking on unsafe sexual behavior and commercial sex. Based on the findings of this study, we were able to identify the adverse effects of street hawking on the social and physical health of young female girls who were involved in street vending. This activity is a reflection of the general economic hardship of low income country as Burkina Faso, and poverty appears to be its primary cause. Thus the adverse effects could be minimized if actions were taken at all levels of government, to identify and manage problems properly. Empowerment of girls might be the proper way of providing protection to them. In addition, systematic surveillance of work related hazards together with education programs should be developed by the government and its partners at hawking places in order to reduce the health risks of the activity.

## Additional file

Additional file 1: Questionnaire_sexual abuse and risky sexual behaviours. (PDF 338 kb)

## Abbreviations

AIDS: Acquired Immune Deficiency Syndrome
AOR: Adjusted Odd Ratio
COR: Crude Odd Ratio
FGM: Female Genital Mutilation
HIV: Human Immunodeficiency Virus
ILO: International Labour Organization
JAFAA: Jonction des Actions pour Faire Avancer l’Afrique
NGO: Non-Governmental Organisation
PAMAC: Programme d’Appui au Monde Associatif et Communautaire
STD: Sexually Transmitted Disease
STI: Sexually Transmitted Infection
WHO: World Health Organization
