# Supplementary material for: Sexual abuse and risky sexual behaviors among young female hawkers in Burkina Faso: a mixed method study
Source: BMC Int Health Hum Rights. 2017 Jan 4;17:1. doi: 10.1186/s12914-016-0109-8 (PMC5209825; doi:10.1186/s12914-016-0109-8)
Supplement: Additional file 1: — Questionnaire_sexual abuse and risky sexual behaviours. (PDF 338 kb) [file 12914_2016_109_MOESM1_ESM.pdf]

QUESTIONNAIRE

SEXUAL ABUSE, RISKY SEXUAL BEHAVIORS AND VULNERABILITY TO OCCUPATIONAL HAZARDS AMONG YOUNG FEMALE HAWKERS IN BURKINA FASO

Questionnaire Number: ………….................

Date of Filling the Questionnaire : ………………………………………

Interviewer's Name : ……………………………………………………………….

Site : (1) Boromo [ ] (2) Bittou [ ]

Greetings! My name is …………………………………. and I am working as an interviewer for the study on Young female Street Vendors. The results of the study will be used for possible interventions among female hawkers. In order to determine what may be appropriate interventions, we are looking into the female hawkers' social conditions, their work and health conditions, their vulnerability for occupational hazards and violence and their sexual behavior.

We have a number of questions that we would like to ask you but please be assured that your responses will be kept in complete confidence. The interview will take approximately 10 to 20 minutes, during which time we will be noting down and recording your answers. Would you have the time to help us with our study?

SECTION A : SOCIODEMOGRAPHIC INFORMATION

1. **Age:** ………
2. **Marital Status:** (1) Single [ ] (2) Married [ ] (3) Concubine [ ] (4) Separated/divorced [ ] (5) Other, specify……………
3. **Educational Level**: (1) Not attend school [ ] (2) Primary [ ] (3) High school [ ] (4) College [ ] (5) University [ ] (6) Informal education [ ]
4. **Current status:** (1) Studying [ ] (2) Not Studying [ ] (3) Other, specify……….
5. **Nationality** (1) Burkinabe [ ] (2) Ghanaian [ ] (3) Togolese [ ] (4)Other, specify……………….
6. **Ethnic group**: (1) Djoula [ ] (2) Mossi [ ] (3) Dagari [ ] (4) Dafi [ ] (5) Samo [ ] (6) Peulh [ ] (7) Bissa[ ] (8) Other, specify…..
7. **Religion :** (1) Muslim [ ] (2) Catholic [ ] (3) Protestant [ ] (4) Other, specify…..
8. **Number of children**: (1) No Child [ ] (2) 1-2 [ ] (3) 3-5 [ ] (4) above 5 [ ]

9. Living situation: (1) Alone [ ] (2) children [ ]

(3) With Husband/Male partner [ ]
Education level : ………….
Occupation: (1) Not working [ ] (2) Trader [ ] (3) Farmer [ ]
(4) Formal job [ ] (5) Other, specify………

(4) With Parents [ ]
Father education level :…….. Father Occupation…….
Mother education level :…….. Mother Occupation….

(5) With Brother/sister [ ]
Education level:………. Occupation :…………….

(6) With Friends [ ]
Education level:………. Occupation :…………….

(7) Other, specify……………
Education level:………. Occupation :…………….

10. Where do you reside (with approximate distance) : …………………………………..

11. Where does your family reside?

(1) With you here [ ] (2) In the Village [ ] (3) Other / Specify …………………………

12. How many members are there in your family?

(1) Less 4 [ ] (2) 4 to 7 [ ] (3) 8 to 10 [ ] (4) More than 11[ ]

SECTION B: HAWKING NATURE / ENVIRONMENT / CONDITIONS

13. Why do you sell on the street (Multiple Coding = MC) ?

(1) Family tradition [ ] (2) Relative forces me [ ] (3) Cannot find other job [ ]
(4) Easy to do [ ] (5) For survival [ ] (6) Feed my family [ ]
(7) Other, specify………………...

14. How often have you been to this area in the last one year?

(1) Almost every day [ ] (2) Occasionally (Please specify) ………………

15. At which age did you start hawking? ……………..

16. When do you usually start hawking each day?

(1) Pre-dawn [ ] (2) Morning [ ] (3) Afternoon [ ] (4) At night [ ]

17. How many hours do you hawk in a day?

(1) 1 to 8 hours [ ] (2) 8 to 12 hours [ ] (3) 12 to 16 hours [ ]
(4) More than 16 hours [ ]

18. Does it happen to you to stay at the trading place over night?

(1) No [ ] (2) Yes [ ] Specify how often ………………

19. Who exactly do you work for?

(1) Self/owner [ ] (2) For family [ ] (3) For employer [ ]
(4) Other (specify)……………….

20. If it is self-owner how much (CFA) do you sell in a day?

(1) Below 5000 [ ] (2) 5000-10000 [ ] (3) Above 10000 [ ]
(4) Other, specify …………………

21. If particular owner, how much wage do you normally receive daily after hawking?

(1) Nothing [ ] (2) Below 1000 [ ] (3) 1000-5000 [ ]
(3) Above 5000 [ ] (4) Other, specify …………………….

22. Do you pay any rent, taxes or levies at your trading location?

(1) No [ ] (2) Yes [ ] Specify …………………………………….

23. How did you raise your capital to start selling?

(1) Not my money [ ] (2) Given to me [ ] (3) Borrowed [ ]
(4) Other, specify ……….

24. Which categories of item do you sell (Multiple Coding)?

(1) Beverages [ ] (2) water [ ] (3) Fruits [ ] (4) Rubber products [ ]
(5) Cookies [ ] (6) Cigarette (7) Food [ ] (8) Other, specify ………

25. Do you have any other work apart from hawking on the street?

(1) No [ ] (2) Yes [ ] Specify …………………………………….

26. Which time do you usually close daily?

(1) If tired [ ] (2) afternoon [ ] (3) Early night [ ]
(4) After 8 pm [ ] (5) No specific time [ ] (6) Other, specify …………

27. How do you spend the money you receive from work (last one week for example)?

(1) Give to parents/guardian [ ] (2) Give to supervisor/Leader [ ] (3) Personal use [ ]
(4) Give part and save part [ ] (5) Save all [ ] (6) Other / Specify………………..

28. Do you belong to any street hawking association?
(1) Yes [ ] (2) No [ ]

29. Are you satisfied with your present job?

(1) Yes, totally [ ] (2) Somewhat [ ] (3) No [ ]

30. In your opinion, how do people view/treat you? (the main answer)

(1) With respect [ ] (2) They mock me [ ] (3) They exploit me [ ]
(4) They ignore me [ ] (5) Other / Specify…………………………………

31. If you are not satisfied or somewhat satisfied, what are the two main reasons for your dissatisfaction (Multiple Coding)?

(1) Lack of safety [ ] (2) Harassment [ ] (3) Difficult conditions[ ]
(4) Low income [ ] (5) Other / Specify……………..

32. Are you aware of any assistance including social, financial support for female hawkers?

(1) No [ ] (2) Yes [ ]

33. If yes who provide it?

(1) Government [ ] (2) NGO [ ] (3)Association [ ]
(4) Others (specify)………………..

34. Have you ever received any assistance?

(1) No [ ] (2) Yes [ ] Specify …………………………………….

SECTION C: WORK PLACE SAFETY

35. Have you ever been sick during the work?

(1) Yes [ ] (2) No [ ]

36. If yes what type of sickness did you have (Multiple Coding)?

(1) Respiratory problem [ ] (2) Contagious disease [ ]
(3) Stomach/intestinal disease [ ] (4) headache [ ]
(5) Back pain due to heavy load [ ] (6) burns [ ] (7) Cuts/wounds [ ]
(8) Others, specify ………………………………………………………………………..

37. What kind of toilet do you currently use?

(1) No toilet [ ] (2) Public Toilet [ ]
(3) Go home [ ] (4) Others (specify)………………………

38. Have you ever been subjected to any of the following while hawking (Multiple Coding)?

(1) Thief [ ] (2) Unpaid goods [ ] (3) Officials' harassment [ ]
(4) Traffic injury [ ] (5) Attack with weapon [ ] (6) Attack without weapon [ ]
(7) Arrest [ ] (8) Confiscations [ ] (9) Others (Specify)……………….

39. Which time do you face more threats at the trading location?
(1) In day time [ ] (2) In night time [ ] (3) Both [ ]

SECTION D : SEXUAL VIOLENCE / HARASSMENT

What kind(s) of sexual harassment/assault have you faced in public (Multiple Coding)?

(1) Verbal (comments, whistling etc) [ ] (2) Visual (staring, leering) [ ]
(3) Physical (touching, feeling up etc.) [ ] (4) Flashing (exhibitionism) [ ]
(5) Stalking [ ] (6) Violent physical attack [ ]
(7) Rape [ ] (8) Misuse of mobile camera [ ] (9) None [ ]
(10) Others (specify)………………………………

How often have you faced such incidents in this area in the past year?

(1) Just once [ ] (2) 2 to 5 times [ ] (3) More than 5 times [ ]
(4) Frequently [ ]

Who harassed you (Multiple Coding)?

(1) Client/customer [ ] (2) Member of the public [ ] (3) Co-hawker [ ]
(4) Driver [ ] (5) Apprentice [ ] (6) Passenger [ ]
(7) Policeman [ ] (8) Park officer [ ] (9) Boyfriend [ ]
(10) Goods' owner [ ] (11) Others (specify)………………………

40. HARASSMENT / ASSAULT

Harassment/Assault	Number of time in this year	Assaulter

41. Think of the most severe sexual harassment / assault you experience, what did you do (MC)?

(1) Nothing [ ] (2) Confronted the perpetrator [ ] (3) Asked bystanders for help [ ]
(4)Told/asked for help from family[ ] (5) Told / asked for help from a friend [ ]
(6) Reported it to the police [ ] (7) Other (specify) ……………………

42. Have you witnessed other hawker being harassed in your work place?

(1) No [ ] (2) Yes [ ] Specify …………………………………….

43. When you see women or girls being harassed in public, what is your reaction?

(1) Support the victim [ ] (2) Get public support [ ] (3) Calling the police [ ]
(4) Prefer not to get involved [ ] (5) Others (specify)……………………

44. How has your family/guardian/boss prepared you to respond to such incidents/experiences?

(1) Not prepared me [ ] (2) Advised me to run away [ ]
(3) Prepared me to deal with such situations [ ] Specify ………………………
(4) Others (specify)……………………………………………….

45. Do you know how to report a threat or a violent incident, and to whom?

(1) No [ ] (2) Yes [ ] Specify ……………………………………..

46. Do you think any of these factors affect women's personal safety in this area (MC)?

(1) Being of a certain age-group (specify………….) (2) Being a woman [ ]
(3) Being disabled [ ] (4) Being of a certain economic class [ ] (5) Being alone[ ]
(6) Being dress less [ ] (7) Working at night [ ] (8) Lack of police [ ]
(9) Men taking alcohol/drug [ ] (10) Poor lighting [ ]
(11) Others (specify)……………………………………………………………………

47. Have procedures for violence prevention been set out for your work area?

(1) No [ ] (2) Yes [ ] Specify …………………………………..

SECTION E: SEXUAL BEHAVIOUR

48. The last time you had sexual intercourse, did you or your partner use a condom?

(1) No [ ] (2) Yes [ ] (3) I have never had sexual intercourse [ ]

49. During the past 3 months, with how many people did you have sexual intercourse?

(1) 1 person [ ] (2) 2 to 4 people [ ] (3) 5 people or more [ ]
(3) I have had sexual intercourse, but not during the past 3 months [ ]

50. How old were you when you had sexual intercourse for the first time?

(1) I have never had sexual intercourse [ ] (2) 14 years old or younger [ ]
(3) 15 years old or older [ ] (4) I don't remember [ ]

51. The last time you had sexual intercourse, what one method did you use to prevent pregnancy?

(1) No method was used [ ] (2)Birth control pills [ ]
(3) Condoms [ ] (4) Depo-Provera (or any injectable birth control) [ ]
(5) Not sure [ ] (6) Others (specify)……………………

52. Have you ever get unwanted pregnant?

(1) No [ ] (2) Yes [ ]

53. If yes what did you do?

(1) Give birth [ ] (2) Clandestine abortion [ ]
(3) Spontaneous abortion [ ] (3) Others (specify)……………………………………

54. Have you ever received money or gift for sexual intercourse?

(1) No [ ] (2) Yes [ ] (3) Decline the offer [ ]

55. Have you ever done your screening for HIV?

(1) No [ ] (2) Yes [ ]

56. Have you ever heard about HIV/AIDS?

(1) Yes [ ] (2) No [ ]

57. Can you the 3 main ways of transmission?

………………………………………………………………..

………………………………………………………………..

………………………………………………………………..

58. Are you sexually mutilated?

(1) No [ ] (2) Yes [ ]

Thank you for your time
